# Biodiversity of *Lactobacillus plantarum* from traditional Italian wines

**DOI:** 10.1007/s11274-014-1654-8

**Published:** 2014-05-10

**Authors:** Bruno Testa, Silvia Jane Lombardi, Patrizio Tremonte, Mariantonietta Succi, Luca Tipaldi, Gianfranco Pannella, Elena Sorrentino, Massimo Iorizzo, Raffaele Coppola

**Affiliations:** 1 Department of Agricultural, Environmental and Food Sciences (DiAAA), University of Molise, via De Sanctis snc, 86100 Campobasso, Italy; 2 School of Agricultural, Forest and Environmental Science, University of Basilicata, Viale dell’Ateneo Lucano 10, 85100 Potenza, Italy

**Keywords:** *Lactobacillus plantarum*, Wine, PCR-DGGE, RAPD-PCR, Malolactic fermentation

## Abstract

In this study, 23 samples of traditional wines produced in Southern Italy were subjected to microbiological analyses with the aim to identify and biotype the predominant species of lactic acid bacilli. For this purpose, a multiple approach, consisting in the application of both phenotypic (API 50CHL test) and biomolecular methods (polymerase chain reaction-denaturing gradient gel electrophoresis and 16S rRNA gene sequencing) was used. The results showed that *Lactobacillus plantarum* was the predominant species, whereas *Lb. brevis* was detected in lower amount. In detail, out of 80 isolates 58 were ascribable to *Lb. plantarum* and 22 to *Lb. brevis*. Randomly amplified polymorphic DNA-polymerase chain reaction was used to highlight intraspecific variability among *Lb. plantarum* strains. Interestingly, the cluster analysis evidenced a relationship between different biotypes of *Lb. plantarum* and their origin, in terms of wine variety. Data acquired in this work show the possibility to obtain several malolactic fermentation starter cultures, composed by different *Lb. plantarum* biotypes, for their proper use in winemaking processes which are distinctive for each wine.

## Introduction

Malolactic fermentation (MLF) is considered a desirable transformation in winemaking processes for the production of some important red wines. It is a deacidification process consisting in the decarboxylation of l-malic acid by the malolactic enzyme and it is a result of the metabolic activity of some lactic acid bacteria (LAB). Nowadays, the use of LAB strains as malolactic starter cultures to improve the wine quality is a common practice in winemaking. *Oenococcus oeni* is probably the best adapted species and it is able to overcome the harsh environmental wine conditions, and therefore this species represents the widespread commercial ML starter culture (Bartowsky and Borneman 2011; Lombardi et al. 2012; Cafaro et al. 2013). However, other LAB species possess many favourable characteristics that would make them suitable candidates for their use as ML starters (du Toit et al. 2011). Among them, several *Lactobacillus* species not only display the ability to survive the harsh wine conditions (Mañes-Lázaro et al. 2009; Izquierdo et al. 2009; Pozo-Bayón et al. 2009; Ruiz et al. 2010), but they also possess enzymes involved in the MLF (Matthews et al. 2007; De Las Rivas et al. 2009). *Lb. plantarum* could be the best candidate for its use in winemaking processes, since it is able to survive under the stress conditions of winemaking (pH 2.8–3.4, alcohol 11–15 %), and to drive the MLF. Moreover, some *Lb. plantarum* strains are able to inhibit spoilage bacteria and to degrade biogenic amines (Capozzi et al. 2010, du Toit et al. 2011). These evidences found confirmation in the practical application of a *Lb. plantarum* strain used as commercial starter culture, recently released by Lallemand, to ensure MLF in musts or wines (Fumi et al. 2010). However, several reports highlighted that the success of MLF starters depends on the used strain and it is influenced by several factors, including the geographical origin of the strain (Gonzàlez-Arenzana et al. 2012), as well as the adaptability to the winemaking processes distinctive for each wine. Moreover, individual strains of *Lb. plantarum* have been found to produce distinctive flavours, and the concentration of some volatile compounds seems to be influenced by the LAB species or the LAB strain, thus reflecting a degree of diversity among strains of the same species (Pozo-Bayón et al. 2005). On these bases, the present work was planned to identify and biotype different *Lb. plantarum* strains naturally occurring in traditional wines from Southern Italy for their next use as proper ML starter cultures in different winemaking processes.

## Materials and methods

### Wine samples

Twenty-three samples of wine were collected from different artisanal wineries located in various areas of Southern Italy. None of the artisanal wineries had ever used LAB commercial starter cultures. One fermentation tank was sampled in each winery when the alcoholic fermentation was completed and the wines underwent spontaneous MLF with the endogenous microbiota. Wine samples were then aseptically taken for physico-chemical and microbiological analyses during MLF.

### Physico-chemical and microbiological analyses

Total acidity, pH and alcohol were determined according to the EC Official Methods (1999). LAB were enumerated and isolated by plating serial decimal dilutions on MRS agar (Oxoid) adding 40 mg/l of cicloheximide to inhibit the yeast growth. Plates were incubated at 28 °C for 72 h under anaerobic conditions using an anaerobic system (Oxoid). Five to ten colonies were picked randomly from MRS plates at the highest dilution having positive growth, excluding those with a number of colonies <30 c.f.u./ml. The purified isolates were maintained frozen at −80 °C in MRS medium with 15 % glycerol.

### Identification

Gram staining, catalase test, microscope observation, study of metabolism (Lafon-Lafourcade et al. 1983), assimilation of carbon sources by the API 50CHL test (bioMérieux), were used to screen the isolates as described by López et al. (2008) and to presumptively identify those belonging to the *Lactobacillus* genus.

Isolates presumptively identified as *Lb. plantarum* were then identified by PCR-DGGE and 16S rRNA gene sequencing and those identified as *Lb. plantarum* were biotyped by RAPD-PCR.

### DNA extraction and purification from pure culture

Two milliliters of each overnight culture was centrifuged at 14,000*g* for 10 min at 4 °C to pellet the cells and the pellet was subjected to DNA extraction according to Querol et al. (1992), with the addition of lysozyme (25 mg/ml, Sigma) and mutanolysin (10 U/ml, Sigma) for bacterial cell-wall digestion. Quantity and purity of the DNA were assessed by optical reading at 260 and 280 nm, as described by Sambrook et al. (1989).

### DGGE analysis

The DNA from each strain was prepared for DGGE by amplifying the V1 region of 16S rRNA using the following primers: P1V1 (5′-GCG GCG TGC CTA ATA CAT GC-3′) (Cocolin et al. 2001) and P2V1 (5′-TTC CCC ACG CGT TAC TCA CC-3′) (Rantsiou et al. 2005). A GC clamp (5′ CGC CCG CCG CGC CCC GCG CCC GTC CCG CCG CCC CCG CCC G-3′) (Sheffield et al. 1989) was attached to the 5′ end of the P1V1 primer. PCR was performed in a Mastercycler gradient (Eppendorf). The reaction mixture (50 µ) consisted of 10 mmol/l Tris–HCl (pH 8.3), 50 mmol/l KCl, 200 µmol/l of each dATP, dGTP, dCTP and dTTP, 1.5 mmol/l MgCl, 0.2 µmol/l of each primer, 200 ng DNA and 1.25 U *Taq*-DNA polymerase (Finnzymes). The amplification program consisted of a 1 min denaturation step at 95 °C, a 1 min annealing step at 45 °C and a 1 min extension step at 72 °C. The first cycle was preceded by an initial step at 95 °C for 5 min. After 35 cycles, there was a final 7 min extension step at 72 °C. Negative controls without DNA template were included in parallel. PCR products were separated in 1.5 % (w/v) agarose gel (Sigma) by electrophoresis for 45 min at 120 V in TBE 0.5 x (Sigma) and were subsequently visualised by UV illumination after ethidium bromide (50 µg/ml) staining (Sigma). PCR products obtained from amplification of V1 region of 16S rRNA were subjected to DGGE analysis, using a DCode Universal Mutation Detection System (BioRad, Hercules, CA, USA). Electrophoresis was performed in a 0.8-mm polyacrylamide gel (8 % [w/v] acrylamide-bisacrylamide [37.5:1]) by using two different ranges of denaturant to optimise separation of the products. Two denaturant gradients, from 40 to 60 % (100 % denaturant was 7 M urea plus 40 % [w/v] formamide) increasing in the direction of electrophoresis run, were used. The gels were subjected to a constant voltage of 120 V for 5 h at 60 °C, and after electrophoresis they were stained for 20 min in 1.25 × TAE containing 50 μg/ml ethidium bromide and visualised under UV illumination. DGGE gels were digitally captured by GEL DOC XR System (Bio-Rad, Hercules, CA, USA) using the software Quantity One Analysis (Bio-Rad) and analysed with the pattern analysis software package, Gel Compare II Version 2.0 (Applied Maths, Kortrijk, Belgium). Calculation of similarities in the profiles of bands was based on Pearson product-moment correlation coefficient. Dendrograms were obtained by mean of the Unweighted Pair Group Method using Arithmetic Average (UPGMA) clustering algorithm (Vauterin and Vauterin 1992).

### Sequence analysis

Two to four representative *Lb. plantarum* strains for each cluster obtained by DGGE analysis were amplified with primers P1 (5′-GCGGCGTGCCTAATACATGC-3′) and P4 (5′-ATCTACGCATTTCACCGCTAC-3′), as described by Klijn et al. (1991), targeting 700 bp of the V1–V3 region of the 16S rRNA gene. After purification, (QIAquick PCR purification kit, QIAGEN GmbH, Hilden), products were sent to a commercial facility for sequencing (Eurofins MWG Biotech Company, Ebersberg, Germany). Sequences were aligned with those in GeneBank with the Blast program (Altschul et al. 1997) to determine the closest known relatives, based on the partial 16S rRNA gene homology.

### RAPD-PCR

Amplification reactions were performed in a 25 µl reaction volume containing 10 mmol/l Tris–HCl (pH 8.3), 50 mmol/l KCl, 200 μmol/l of each dATP, dGTP, dCTP and dTTP, 1.5 mmol/l MgCl2, 1 μmol/l primer, 80 ng DNA and 1.25 U *Taq*-DNA polymerase (Finnzymes, Finland). A Mastercycler gradient (Eppendorf, Hamburg, Germany) was used with the following primers and amplification conditions: (a) M13: 5′GAGGGTGGCGGTTCT3′ (Huey and Hall 1989); the amplification was carried out for 35 cycles of 94 °C for 1 min, 40 °C for 20 s, ramp to 72 °C at 0.5 °C/s, 72 °C for 2 min; (b) D8635: 5′-GAGCGGCCAAAGGGAGCAGAC-3′ (Akopyanz et al. 1992); after an initial step of 94 °C for 2 min the amplification was performed for 35 cycles of 94 °C for 1 min, 42 °C for 1 min, 72 °C for 1 min and 30 s, and a final step at 72 °C for 10 min.

The amplification products were separated by electrophoresis on 1.5 % (w/v) agarose gel (Sigma-Aldrich, Steinheim, Germany) in 0.5 x TBE buffer and then subjected to ethidium bromide staining. RAPD-PCR gels were digitally captured and analysed as previously described for DGGE analysis.

## Results

### Physico-chemical and microbiological analyses of wine

The physico-chemical and microbiological features of wine samples are reported in Table 1. Samples were characterized by pH values ranging from 3.54 (sample TI1) to 3.88 (sample AG1). These values comply those of the typical wines traditionally produced in Southern Italy (Gambuti et al. 2007; Suzzi et al. 2012). The highest alcohol levels were appreciated in Aglianico, Taurasi and Tintilia samples, while the highest levels of acidity were detected in Tintilia and Montepulciano samples.

**Table 1:** Physico-chemical and microbiological features of 23 traditional red wine samples from Southern Italy

Sample	Type of wine	Localities	pH	Alcohol	l-Malic acid^a^	l-Lactic acid^a^	MRS^b^
AG1	Aglianico	Campania	3.88	13.6	0.1	2.3	6.5 × 10^4^
AG2	Aglianico	Campania	3.71	13.6	0.8	2.1	5.0 × 10^4^
MT1	Montepulciano	Molise	3.68	13.5	0.9	1.9	6.0 × 10^3^
MT2	Montepulciano	Molise	3.70	13.3	0.6	1.4	4.0 × 10^3^
MT3	Montepulciano	Molise	3.65	11.2	0.5	2.2	3.3 × 10^3^
MT4	Montepulciano	Molise	3.60	11.2	1.2	2.4	5.5 × 10^5^
MT5	Montepulciano	Molise	3.79	11.8	1.1	2.5	5.8 × 10^5^
MT6	Montepulciano	Molise	3.80	11.8	1.4	2.3	4.8 × 10^6^
PI1	Pentro d’Isernia	Molise	3.77	11.3	1.3	1.8	2.4 × 105
PI2	Pentro d’Isernia	Molise	3.76	11.6	0.7	1.6	2.2 × 10^3^
PD1	Piedirosso	Campania	3.68	12.6	0.4	1.9	7.8 × 10^3^
PD2	Piedirosso	Campania	3.62	12.4	0.6	1.8	7.5 × 10^3^
PD3	Piedirosso	Campania	3.65	12.8	0.4	2.1	6.8 × 10^3^
RM1	Rosso Molise	Molise	3.62	12.5	0.9	1.6	9.8 × 10^3^
RM2	Rosso Molise	Molise	3.80	12.1	1.6	2.3	1.2 × 10^5^
TA1	Taurasi	Campania	3.76	14.2	1.5	1.8	2.3 × 10^3^
TA2	Taurasi	Campania	3.69	14.1	1.4	1.9	4.3 × 10^3^
TI1	Tintilia	Molise	3.54	14.6	0.2	1.9	8.8 × 10^5^
TI2	Tintilia	Molise	3.86	14.2	0.4	2.2	3.4 × 10^5^
TI3	Tintilia	Molise	3.76	14.0	0.6	1.9	4.5 × 105
TI4	Tintilia	Molise	3.86	14.3	0.7	1.5	2.8 × 10^4^
TI5	Tintilia	Molise	3.80	14.0	0.3	1.3	6.6 × 10^5^
TI6	Tintilia	Molise	3.70	14.4	0.8	1.8	8.9 × 10^4^

^a^g/l; ^b^ c.f.u./ml

Microbiological analyses evidenced the presence of lactic acid bacteria (LAB) at levels ranging from 2.2 × 10^3^ c.f.u./ml (sample PI2) to 4.8 × 10^6^ c.f.u./ml (sample MT6). The differences in physico-chemical and microbiological parameters appreciated in this study are common in wines, also deriving from the same geographical area, since several factors, including the grape variety, the age of wines, the environmental conditions can influence the wine features (du Toit et al. 2011).

### Phenotypic and molecular identification

**Fig. 1:**
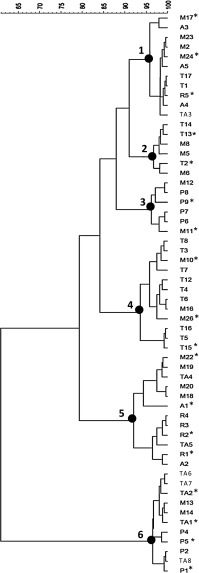
Dendrogram showing PCR-DGGE profiles of 58 lactobacilli isolated from 23 traditional red wine samples from Southern Italy

**Fig. 2:**
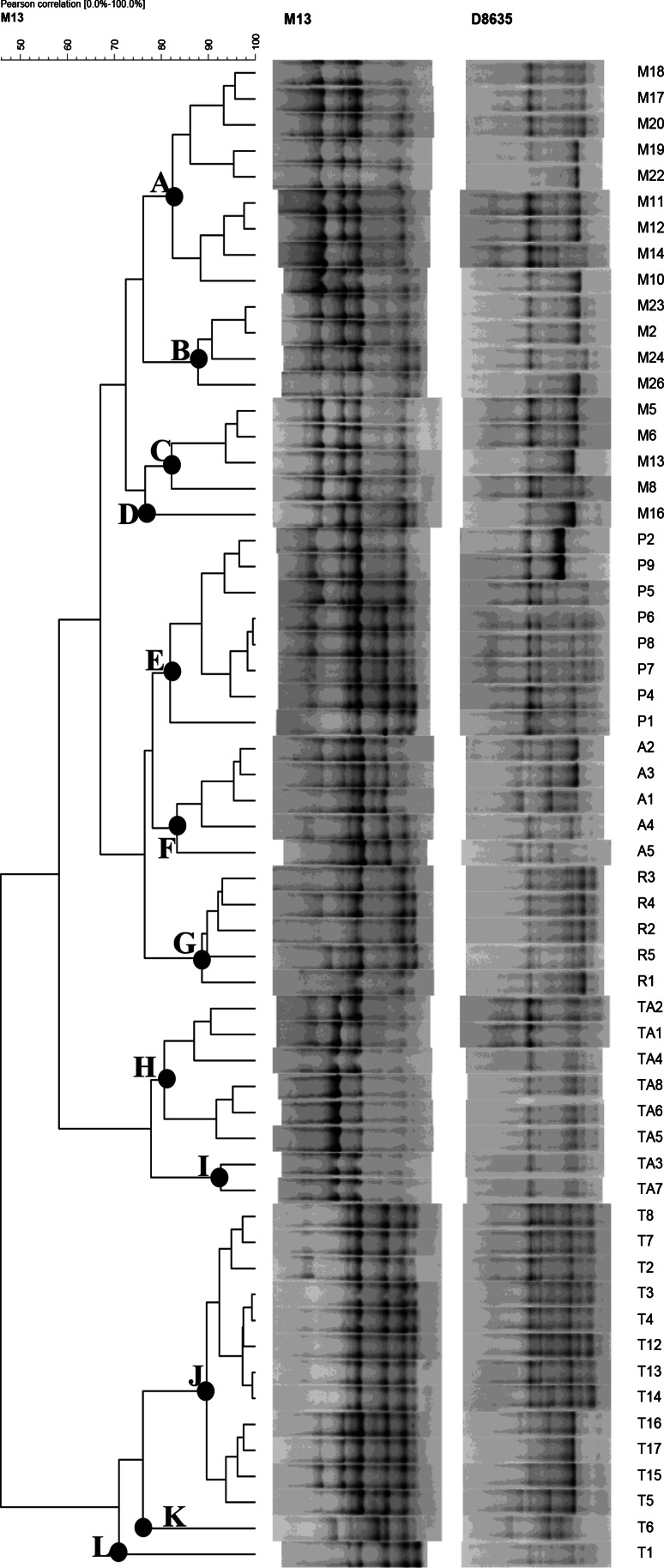
Dendrogram showing RAPD-PCR profiles of 58 *Lb. plantarum* strains isolated from 23 traditional red wine samples from Southern Italy

Out of 184 isolates, 80 Gram positive, catalase-negative and rod-shaped microorganisms were presumptively identified as lactobacilli and were subjected to API 50CHL identification. According to the species description in Bergey’s Manual (Kandler and Weiss 1986), the phenotypic results highlighted that 22 isolates were ascribable to *Lb. brevis*, and 58 to *Lb. plantarum* (Table 2). However, some doubts were raised for the identification of 4 *Lb. plantarum* strains: API 50CHL profiles suggested the assignation to this species at only 62 % similarity level. The real identity of the 58 presumptive *Lb. plantarum* strains was confirmed by PCR-DGGE analysis (Fig. 1). The strains were grouped according to the migration profiles into 6 clusters. For each cluster, 2–4 strains were subjected to sequencing for identification purposes. The results of the sequencing (Table 3) allowed the identification of all the 18 selected strains. Combining these results with those obtained from the DGGE cluster analysis, it was possible to identify all the 58 strains as *Lb. plantarum*, which were subsequently characterised through RAPD-PCR analysis (Fig. 2). On the basis of RAPD-PCR band profiles, the assayed *Lb. plantarum* strains were divided into 12 clusters. Clusters A (9 strains), B (4 strains), C (4 strains), and D (1 strain) grouped all the strains isolated from Montepulciano wines; all the 8 *Lb. plantarum* strains from Piedirosso and Pentro d’Isernia wines were grouped into cluster E; the 5 strains from Aglianico wines were grouped into cluster F, and those (5 strains) from Rosso Molise into cluster G; clusters H and I grouped 8 *Lb. plantarum* strains from Taurasi wines, and clusters J, K and L those (14 strains) from Tintilia wines.

**Table 2:** Preliminary identification and API 50CHL identification of 80 lactobacilli isolated from 23 traditional red wine samples from Southern Italy (strains were grouped on the basis of similar API profiles)

Wine origin	Number of strains	Preliminary identification^a^	Identification by API50 CHL	Quality of identification by API50 CHL
MT, PD, TA	6 strains	*Lb. plantarum*	*Lb. plantarum*	Excellent
AG, MT, TI	7 strains	*Lb. plantarum*	*Lb. plantarum*	Excellent
MT, TA	4 strains	Doubtful	*Lb. plantarum*	Doubtful
AG, MT, TA	5 strains	*Lb. plantarum*	*Lb. plantarum*	Excellent
MT, PD	5 strains	*Lb. plantarum*	*Lb. plantarum*	Excellent
MT, RI	6 strains	*Lb. plantarum*	*Lb. plantarum*	Excellent
RM, TI, TA	8 strains	*Lb. plantarum*	*Lb. plantarum*	Excellent
MT, PD, PI, TI	8 strains	*Lb. plantarum*	*Lb. plantarum*	Excellent
MT, TI, TA	11 strains	*Lb. plantarum*	*Lb. plantarum*	Excellent
MT, PI, PD	4 strains	*Lb. brevis*	*Lb. brevis*	Excellent
MT, PD	3 strains	*Lb. brevis*	*Lb. brevis*	Very good
PI, PD, RM	4 strains	*Lb. brevis*	*Lb. brevis*	Good
AG, MT	3 strains	*Lb. brevis*	*Lb. brevis*	Excellent
RM, PI	4 strains	*Lb. brevis*	*Lb. brevis*	Very good
AG, MT, PD	4 strains	*Lb. brevis*	*Lb. brevis*	Good

^a^The preliminary identification was obtained by Gram staining, catalase test, microscope observation and study of metabolism

**Table 3:** Identification, based on blast comparison in GenBank, of 18 lactic acid bacilli selected on the basis of DGGE cluster analysis

Cluster	Strain	Size	Closest relative	% Identity	Source^a^
1	M17	664	*Lb. plantarum*	100	GU138574
	M24	584	*Lb. plantarum*	100	GQ922601
	R5	635	*Lb. plantarum*	99	JQ278711.1
2	T13	634	*Lb. plantarum*	100	JF728278.1
	T2	636	*Lb. plantarum*	100	JF728278.1
3	P9	636	*Lb. plantarum*	100	JQ278711.1
	M11	635	*Lb. plantarum*	100	JQ278711.1
4	M10	667	*Lb. plantarum*	99	FJ915780
	M26	650	*Lb. plantarum*	99	GU138574
	T15	616	*Lb. plantarum*	99	JQ278711.1
5	M22	481	*Lb. plantarum*	99	JQ278711.1
	A1	634	*Lb. plantarum*	100	GU138574
	R2	636	*Lb. plantarum*	100	JQ278711.1
	R1	634	*Lb. plantarum*	100	JQ278711.1
6	TA2	635	*Lb. plantarum*	99	AB112083.1
	TA1	637	*Lb. plantarum*	99	AB112083.1
	P5	611	*Lb. plantarum*	99	GU299081.1
	P1	611	*Lb. plantarum*	99	GU299081.1

^a^Accession number of the sequence of the closest relative found by blast search

## Discussion

Results of physico-chemical and microbiological analyses evidenced that red wines traditionally produced in Southern Italy are characterised by low pH values and high alcohol levels, both contributing to the creation of an adverse environment where only few species of lactic acid bacilli are able to survive. Among them, high resistant species are able to form colonies on culture media, whereas others enter in a viable but non culturable state, as a response to environmental stresses (Divol and Lonvaud-Funel 2005). In our work, *Lb. plantarum* was the main species isolated from MRS agar plates, and this fact underlines its high tolerance to low pH and high alcohol content. In detail, *Lb. plantarum* was found in association with *Lb. brevis* in Aglianico, Montepulciano, Pentro d’Isernia, Piedirosso and Rosso Molise wines, whereas it was the sole species found in Tintilia and Taurasi wines, characterised by the highest alcohol level (Francesca et al. 2014; Iorizzo et al. 2014).

The results obtained in this study underline the usefulness of molecular methods to describe the presence and biodiversity of *Lb. plantarum* in traditional wines. In detail, if PCR-DGGE analysis and 16S rRNA gene sequencing can be considered by now suitable tools to identify lactobacilli from wines (Bokulich et al. 2012; Ivey and Phister 2011), RAPD-PCR technique revealed an unexpected biodiversity among *Lb. plantarum* strains isolated from different wines. In fact, at the least 12 different biotypes were individuated, and a relationship between the different *Lb. plantarum* biotypes and their origin, in terms of wine type, was observed.

Different Authors already emphasized the predominance of *Lb. plantarum* after alcoholic fermentation and during MLF in wine samples (Beneduce et al. 2004; Spano et al. 2007; Ruiz et al. 2010). Based on these evidences, the application of *Lb. plantarum* as co-inoculant in grape must or as inoculant after alcoholic fermentation should be promoted, not only because of its ability to survive under wine conditions, but also for the ability of certain suitable strains to bring correctly the biological deacidification of red wines. In this connection, it is significant that many wine-associated *Lb. plantarum* strains are equipped with genes encoding for the enzymes involved in the MLF and several enzymes are active under winemaking conditions (Grimaldi et al. 2005; De las Rivas et al. 2009; du Toit et al. 2011). Also, *Lb. plantarum* shows a more diverse enzymatic profile than *O. oeni* (Matthews et al. 2007; Mtshali et al. 2010), and some Authors suggested that this feature could play an important role in the modification of the wine aroma profile (Swiegers et al. 2005; Lerm et al. 2011).

Previous findings suggest that *Lb. plantarum* based starter cultures for MLF in traditional wines can be properly formulated considering the wine-type and/or its geographical area of origin, so the results obtained in the present study represent the starting point to select different biotypes of *Lb. plantarum* that will be assayed for their specific technological attitude for each wine type. Moreover, the diversity of *Lb. plantarum* strains associated with the wine-type suggests their potential application as fingerprinting tools to ensure the traceability and the authentication of traditional red wine. This last topic represents a crucial issue that, in the last years, stimulated the interest of both producers and researchers to protect wines from adulteration practices (Kokkinofta et al. 2014).
